# Grounding systems enhancement using biodegradable waste soil additives: Measurement and analysis

**DOI:** 10.1371/journal.pone.0355934

**Published:** 2026-08-12

**Authors:** Salem Mgammal Al-Ameri, Waleed M. Hamanah, Ali Ahmed Salem, Mohd Fairouz Yousof, Fadhl S. N. Alsarahi, A. Abu-Siada

**Affiliations:** 1 Interdisciplinary Research Center for Sustainable Energy Systems, King Fahd University of Petroleum and Minerals, Dhahran, Saudi Arabia; 2 Department of Electrical Power Engineering, Faculty of Electrical and Electronic Engineering, University Tun Hussein Onn Malaysia, Batu Pahat, Johor, Malaysia; 3 Department of Mathematics, Saber Faculty of Applied Sciences and Humanities, University of Aden, Aden, Yemen; 4 Electrical and Computer Engineering Discipline, Curtin University, Bentley, Western Australia, Australia; Monash University, AUSTRALIA

## Abstract

A grounding system is essential for ensuring the safety and reliability of structures, buildings, telecommunication facilities, and transmission line towers by protecting them from short faults or lightning strikes during storms. An efficient grounding system requires low-resistance pathways, which can be achieved by incorporating grounding enhancement materials as additives to the local soil. Grounding enhancement materials generally include conventional chemical agents, natural substances, and modern waste-derived alternatives. This paper proposes the use of new biodegradable waste materials and a metrological framework to improve the effectiveness of grounding systems. In this regard, five grounding system installations were developed and tested using locally available materials in Miri, Sarawak, Malaysia. Investigated materials as high to lower performance are include palm oil decanter cake (DC), wash ash mixed with sea sand (WS, 50:50), coconut shell charcoal (CC), and palm oil mesocarp fibre (POMF). For comparison, the systems were monitored over 6 months from 15 October to 30 April the nest year using the fall-of-potential (FOP) method measurement technique with the 62% rule of single-rod and layered low-resistivity-material (LRM) configurations. This refines a measurement technique that was compared to the performance of the system with native soil (NS). A post-installation proposed for boosting treatment (activated-carbon slurry for CC; decanter-cake slurry for DC) restored resistance improvements over a short observation. The findings contribute to measurement science by finding that validated biodegradable soil-enhancement materials significantly improve grounding resistance over time. Particularly, the CC and DC offer a low-cost, friendly, environmentally responsible option for enhancing grounding system resistance.

## Introduction

Lightning strikes occur when there is an imbalance of electric charges between clouds and the ground, resulting in a natural atmospheric discharge. This phenomenon can also happen between storm clouds. The lightning strikes flash within a few microseconds and cannot be prevented or stored. Lightning poses a serious hazard to structures, people, animals, and equipment. It is estimated that there are approximately 1.46 billion lightning strikes per year worldwide, which is equivalent to an average of 46 strikes per second, or nearly 2.9 strikes per square kilometer [1]. Therefore, an effective grounding system is essential to protect the overall environment and buildings by redirecting the high current generated by lightning strikes into the earth [2]. The lightning protection system consists of three main parts: a lightning terminator to intercept the discharge, a down conductor to guide the current to the ground, and a grounding device to absorb the transient energy and dissipate it into the surrounding soil. To achieve this, the grounding system must be designed to maintain the lowest possible grounding resistance [3].

In this regard, the performance of a grounding system depends mainly on the soil resistivity (additive soil) at the installation site. In areas with poor soil conditions, enhancement materials and techniques are used to lower grounding resistance and improve current dissipation during fault and lightning events. In conventional grounding practices, engineers enhanced system performance by increasing electrode depth or diameter, interconnecting multiple rods, or installing grounding grids to expand the soil contact area. Chemical treatments such as bentonite, conductive concrete, and salt are also widely used to increase soil conductivity [4–9]. However, these methods often pose challenges related to durability, environmental impact, and maintenance. Ongoing research is exploring sustainable alternatives for improving grounding resistance [4]. This paper emphasizes the application of biodegradable additives as a novel and sustainable strategy for improving grounding system performance, with a focus on balancing technical effectiveness and environmental sustainability.

Among natural soil materials, bentonite, a common clay found in East Asia, has long been used as an effective means of improving soil resistivity in grounding systems. In [10], Several soil enhancement compositions were examined, including pure bentonite, pure wood ash, pure fly ash, and mixtures of bentonite–wood ash, bentonite–fly ash, and wood ash–fly ash. The results showed that the combination of bentonite and wood ash was the most effective, reducing the grounding resistance by up to 80%. Another study has investigated the use of coconut shell in combination with bentonite for improving grounding systems [11]. The results showed that bentonite was more effective than coconut shell in reducing grounding resistance. Incorporating bentonite into laterite and peat soils proved to be beneficial. However, its effectiveness is significantly affected by environmental conditions, as soil moisture content and temperature can substantially affect resistivity measurements. Dry conditions and high temperatures can reduce its performance, causing resistance values to deviate from expected results [9].

The natural environment of the Middle East is dominated by desert terrain, which is associated with high soil resistivity. To address this challenge, researchers evaluated the effectiveness of bentonite as a grounding enhancement material at 11 different locations, examining the relationship between soil resistivity and the number, size, and depth of grounding electrodes [12]. In addition to bentonite, researchers evaluated several natural materials, including plantation clay, rice flour, and coconut shell peat. Among these, plantation clay demonstrated the greatest improvement, achieving the highest percentage reduction in grounding resistance, and proved to be a promising complementary additive alongside bentonite [13]. A recent survey [14] indicated that grounding systems using only bentonite showed the most effective performance. The study compared pure bentonite, pure peat moss, and two different ratios of bentonite-peat moss mixtures. An earlier study [15] also examined the use of other natural reinforcement materials, including zeolite, perlite, and vermiculite. The zeolite was considered the most suitable agriculturally based material for grounding systems due to its excellent water retention capacity and consistent performance across different soil types and climatic conditions. Another study [16] compared various bentonite mixtures with silica sand, China clay, laterite, and bauxite. The results showed that the combination of bauxite and bentonite increased the breakdown voltage by 41% compared to the other samples. Additionally, bauxite has been recognized as a practical and portable grounding material, particularly effective in areas with high soil resistivity, such as rocky terrain, forested areas, and arid desert environments [16].

Chemical enhancement materials generally require less maintenance than traditional methods, as the use of conductive backfill improves the stability and overall reliability of electrical installations [17]. An extensive two-year study investigated the effectiveness of several low-cost materials, including lime, coke powder, metal oxide powders, and sodium chloride, in reducing ground resistance [17]. The study concluded that the metal oxide powders exhibited the most stable long-term performance; at the same time, sodium chloride was considered unsuitable due to its high corrosiveness and tendency to cause large fluctuations in electrode resistance. Similarly, a study conducted by a Saudi Arabian university in 2010 focused on developing low-resistivity backfill materials for grounding applications. The materials tested included bentonite, water, NaSO₄, MgSO₄, MgCO₃, and Na₂CO₃. The results showed that bentonite, NaSO₄, and MgSO₄ were particularly effective in significantly reducing ground resistance [18]. Further research explored different types of bentonites, namely natural bentonite, sodium bentonite, and calcium bentonite, as potential materials for enhancing grounding system performance [19]. Another study investigated the use of bentonite-incorporated concrete as a grounding reinforcement material, demonstrating its effectiveness in reducing grounding resistance. Bentonite was incorporated into the concrete in varying proportions, with the cement content ranging from 35% to 40%. The lime component consisted of 40%–50% alumina, up to 15% iron oxide, and typically less than 6% silica, with calcium aluminate as the primary constituent. The bentonite content in the tested mixtures ranged from 10% to 60% and up to 70%, with the 20% mixture producing the lowest resistance value [20]. The study recommended further research on combining bentonite with conductive concrete and natural soils, particularly in rocky terrains, to optimize grounding performance [21].

Sodium chloride (NaCl) has been a widely used chemical for improving grounding resistance. An experimental study evaluated the effectiveness of several salts, namely sodium chloride (NaCl), magnesium chloride (MgCl₂), sodium thiosulfate (Na₂S₂O₃), and ammonium chloride (NH₄Cl) by measuring grounding resistance over a period of 141 days. While initial changes were observed during the first three weeks, the measurements stabilized thereafter. The results confirmed that all tested chemical salts significantly reduced grounding resistance, with sodium chloride outperforming ammonium chloride. Consequently, NaCl was identified as the most effective soil additive among the salts tested [22].

The waste materials have also been utilized as additive components in grounding systems. Research has examined the use of industrial by-products as soil reinforcement materials for grounding applications. One study [23] investigated gypsum waste obtained from a civil construction recycling facility and fly ash sourced from a coal-fired thermal power plant. Experimental results revealed that the combination of gypsum fragments and fly ash improved the soil’s electrical conductivity and reduced grounding resistance. However, in sandy soils with high porosity, heavy rainfall can cause metal ions from the fly ash to leach into deeper soil layers, thereby decreasing electrical conductivity and potentially impacting the surrounding environment. Other study in [24] have also examined the use of waste-derived materials such as fly ash and biochar to enhance the performance of the grounding system. The findings revealed that a mixture containing 25% fly ash and 75% biochar significantly reduced ground rod resistance, achieving a reduction of up to 88.5% at a test frequency of 50 kHz. Additionally, wood ash and spent activated carbon were evaluated as grounding enhancement materials, with results showing that both wood ash exhibited superior water retention capabilities compared to other alternatives [25]. Other studies have evaluated the potential of locally available conductive by-products such as tire ash and palm kernel oil cake. Among these materials, tire ash exhibited the lowest and most stable resistance values throughout the monitoring period, demonstrating its reliability as an effective additive for improving grounding performance [26]. Similarly, palm kernel oil cake was found to effectively reduce grounding resistance and maintain this improvement over an extended period [27]. An experimental study conducted by the National Defense University of Malaysia [28] explored the use of rice straw ash, sugarcane bagasse ash, Himalayan pink salt, and bamboo salt as grounding enhancement materials. Rice straw, an agricultural byproduct left after grain harvesting, can be converted into ash through combustion. Depending on the combustion process, two types of rice straw ash may be produced: white ash and black ash, both characterized by very low resistivity, making them suitable for grounding applications. Likewise, sugarcane bagasse, a residue from sugarcane processing composed of approximately 50% cellulose, 25% hemicellulose, and 25% lignin, has shown similar potential. The study results demonstrated that both rice straw ash and sugarcane bagasse ash can serve as effective backfill materials, significantly reducing soil resistivity and thereby improving grounding system performance.

Another approach for grounding enhancement involves soil treatment. After a grounding system is installed, its resistance may increase over time due to factors such as rising soil temperature, excessive moisture content, and increased salinity, all of which can negatively affect soil resistivity. An ineffective grounding system not only reduces performance but also heightens the risk of electric shock. A study reported in [29] investigated the use of saltwater for soil treatment following the installation of grounding systems. The experiment, conducted at two different locations, involved applying a saltwater solution around the grounding rods and measuring resistance after 24 hours. The results showed that areas with higher salt concentrations experienced a substantial decrease in resistivity up to 75%. In a related study, agricultural by-products such as rice straw ash and sugarcane bagasse ash were also used as soil treatment agents to reduce grounding resistance. Furthermore, salts such as bamboo salt and Himalayan pink salt were tested in combination with these waste materials, and the findings confirmed that both significantly reduced soil resistivity [28].

Although traditional methods and chemical treatments have been widely used to reduce grounding resistance, they often pose environmental and cost-related challenges. In regions such as Sarawak, Malaysia, where soil conditions are highly variable and often exhibit elevated resistivity, there is a pressing need for sustainable and locally adaptable solutions. According to standards established by the National Fire Protection Association (NFPA) and the Institute of Electrical and Electronics Engineers (IEEE), acceptable grounding resistance should not exceed 5.0 ohms [29,30]. The methods of performing the measurement are in the next section, and they are highlighted in references [31–39]. The comprehensive review of the previous additive soil enhancement is summarized in Table 1.

**Table 1:** Review of methods for grounding resistance improvement.

Ref.	Year	Research Region	Enhancement Material	Type	Advantages	Limitations
[27]	2009	Ghana	Palm kernel oil cake	Waste	Sustained resistance reduction over time.	Seasonal variations are not considered.
[13]	2010	Malaysia	Coconut coir, clay, paddy dust, Bentonite	Waste and natural	Cost-effective; stable for 138 days; paddy dust performed best.	Longer-term validation required.
[17]	2010	Sri Lank	NaCl, metal oxide, lime, coke breeze	Chemical waste	Metal oxide is effective, low-cost, and corrosion-resistant.	Sensitive to moisture; NaCl is unsuitable.
[18]	2010	Saudi Arabia	Bentonite + salt + water	Natural and chemical	Sulfates significantly reduced resistivity.	Avoid mixing bentonite with NaCl.
[22]	2010	Malaysia	NaCl, Na2SO3, MgCl2	Chemical	NaCl is most effective after 141 days.	Potential rod degradation; corrosion risk.
[20]	2012	Malaysia	Bentonite-mixed concrete	Natural and chemical	The 20% mix yielded the lowest resistance.	Longer testing duration needed.
[21]	2012	Greece	Bentonite, conductive concrete, natural soil	Natural and chemical	Cost-effective grounding enhancement.	Requires 3–5 years of field testing.
[19]	2013	Malaysia	Sodium- and calcium-bentonite	Natural and chemical	Sodium bentonite showed superior performance.	Limited test duration.
[15]	2017	Malaysia	Zeolite, Perlite, Vermiculite	Natural	Zeolite is identified as the most effective agricultural additive.	Perlite has low density; vermiculite is less permeable.
[28]	2017	Malaysia	Rice straw ash, bagasse ash, bamboo salt	Waste and natural	Rice straw ash performed best.	Bamboo salt is costly.
[10]	2018	Malaysia	Bentonite, Fly ash, Wood ash	Natural and waste	Up to 80% reduction in grounding resistance; low cost.	Fly ash is found unsuitable.
[33]	2018	Malaysia	Mixture of natural materials	Natural	Affordable; slight reduction in soil resistivity.	Weather conditions affect performance.
[40]	2018	Malaysia	Paddy Husk Ash	Waste	Copper electrodes showed higher durability.	Paddy husk ash is ineffective as an additive.
[11]	2019	Malaysia	Bentonite and coconut husk	Natural and waste	Bentonite yielded the lowest resistance.	Coconut husk is less effective.
[32]	2024	Malaysia	Hydrogel, silica gel, charcoal ash	Chemical and waste	Effectively lowers resistance.	Requires long-term morphology and stability studies.
[24]	2022	Indonesia	Biochar and fly ash	Waste	Achieved 89.5% resistance reduction.	Test duration not specified.
[14]	2023	Malaysia	Bentonite and peat moss	Natural	100% bentonite showed the best performance.	Moisture absorption behavior noted.
[26]	2020	South Africa	Tyre ash, palm kernel oil cake	Waste	Consistent resistance over time.	Tyre ash may leach contaminants.
[38]	2020	Japan	Concrete + bentonite + coconut fibre	Chemical, natural, waste	Significantly lowers resistance.	Urban and climatic impact unassessed.
[41]	2020	Malaysia	Kenaf, straw rice ash, palm oil residue	Natural and waste	High reduction due to optimized particle size.	Requires extended testing (60 + days).
[42]	2019	Indonesia	Gypsum	Natural	Absorbs moisture; reduces resistance.	May produce hydrogen sulfide gas.
[25]	2024	Egypt	Waste active carbon, wood ash	Waste	High water retention; improved safety.	Requires further evaluation for sustainability.
[43]	2023	Indonesia	Rice husks + salt (NaCl)	Waste and chemical	90% resistance is reduced (biopower approach).	Organic degradation over time.
[44]	2021	Iraq	Bentonite	Natural	20% reduction in resistance.	Importation challenges for local use.
[23]	2020	Brazil	Coal ash, plaster waste	Waste	Enhanced conductivity; reduced environmental impact.	Needs economic feasibility analysis.
[45]	2016	Malaysia	Bentonite and sand	Natural	Bentonite outperforms sand.	Risk of fulgurite formation.
[46]	2016	Eswatini	Bentonite, conductive cement	Natural and chemical	Reduced grounding impedance.	Lightning performance not evaluated.
[12]	2016	Middle Est	Bentonite and other GEM	Natural and chemical	Cost-effective across multiple sites.	Difficult backfill application.
[16]	2021	Pakistan	Bauxite, laterite, China clay, etc.	Natural	Low resistance in wet/dry conditions.	Requires mineral extraction processing.
[47]	2022	Malaysia	clay mixture	Natural	Clay is mixed with kenaf	Simulation based

This study addresses this need by experimentally evaluating biodegradable, waste-derived additives as alternative backfill materials for enhancing grounding resistance. Five grounding system models were developed and tested, with resistance measured over an extended monitoring period using the fall-of-potential (FOP) method. The results were compared against those obtained from a conventional local soil system. By integrating biodegradable and low-resistivity with higher carbon content waste materials, this study aims to improve soil conductivity, lower grounding resistance, and establish an environmentally friendly, cost-effective, and reliable grounding system suitable for standardization in future construction projects across Sarawak. Ultimately, the proposed approach contributes to enhancing the safety, resilience, and sustainability of electrical installations.

The research gap and the novelty of this research explicitly distinguish this work from previous studies by highlighting:

- This research evaluates the locally biodegradable waste materials for grounding enhancement and uses, for the first time, the palm oil mesocarp fibre as a novel grounding enhancement material compared to previous studies in [10–15,19–22,28,32,33,40,41,45,47].
- This study experimentally investigates the long-term field monitoring of the actual grounding system at approximately seven months to experience different aspects regarding seasonal variability and whether changes that were not considered in previous research. Most previous research has focused on short-term performance evaluations and on conventional enhancement materials such as bentonite in [12,18–21,45,46].
- This study develops an assessment of the post-instillation soil additive treatment technique using the biodegradable waste-derived materials.

In Table 1, the limitations of previously used additive materials in the literature are highlighted, such as high procurement cost, environmental unfriendliness, corrosiveness to the electrode, and limited availability of materials. The chemical additives are harming the surrounding soil, as in [17,22,32,38]. This additive is suggested to cause environmental issues and corrosion risk. The long-term study in [21] shows low cost-effectiveness of using Bentonite, conductive concrete, and natural soil. Based on available and low-cost biodegradable materials in Malaysia, Sarawak, the CC, DC, WS, and POMF were selected, as supported by research in [4,9,25,51–53,54]. But in these previous studies, they do not provide a long-term and different system of palm oil waste, such as POMF. Also, there is no available plane for sustainable grounding enhancement materials. Limited long-term field investigations have been reported on the performance and degradation behavior of these materials under tropical environmental conditions.

## Methodology and measurement setup

**Fig 1:**
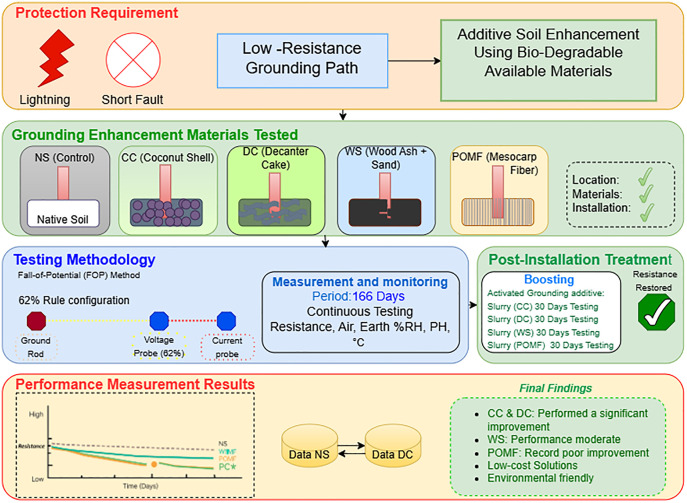
The graphical Framework for the evaluation study of biodegradable grounding enhancement material systems.

The framework of the overall work on grounding enhancement using the biodegradable materials is shown in Fig 1. Several techniques are available for measuring the resistance of grounding systems, each based on distinct measurement principles and offering varying levels of accuracy. In addition, the design and configuration of the grounding electrode play a crucial role in measurement outcomes. The primary objective of these evaluations is to ensure that the grounding system is constructed and maintained to provide safe, reliable, and efficient operation.

### Measurement methods

The two-point method is one of the simplest techniques for measuring ground resistance. It employs two electrodes: one to inject current into the soil and the other to measure the resulting voltage drop. In practical applications, the resistance of the measuring electrode is generally negligible compared to that of the surrounding soil. Although this method is straightforward and quick to implement, its accuracy decreases when used for low-resistance grounding systems [6].

The fall-of-potential (FOP) method, also known as the three-point or three-pole method, is widely regarded as the standard technique for measuring electrode resistance. It is commonly applied in single- or double-rod configurations and other small electrode systems. The setup involves three soil contact points: a test electrode connected via a green wire, a current electrode connected via a red wire placed at a distance from the test electrode, and a potential electrode connected via a yellow wire. This configuration minimizes mutual interference, enabling accurate resistance measurements. The FOP method is frequently used to assess soils treated with reinforcement materials, where changes in measured resistivity serve as indicators of performance improvement [19,31]. For instance, one study applied this method to evaluate the effects of hydrogel, silicone gel, and carbon ash as backfill materials, finding that hydrogel achieved the greatest reduction in grounding resistance, lowering it to 56% of the reference system [32]. Overall, the FOP method is considered the most accurate approach for monitoring grounding system resistance.

In this study FOP method has been used to measure the grounding resistance for over 6 months. The technical specifications of the Grounding resistance measurement device are presented in Table 2. The table summarizing the accuracy range, and resolutions. The Digital Earth Tester can measure both the earth resistance and voltage. The accuracy of the measured resistance is at ±2%. The accuracy has been measured by measuring the earth resistance at different distances (5m, 10m) and different directions (South, North). This practice is standard in the earth resistance measurement to monitor the errors in measurement. Before measuring the earth resistance, the earth voltage test should be 0V then we can obtain the resistance measurement.

**Table 2:** Technical specifications of the grounding resistance measurement device.

Device Name	Purpose	Range	Accuracy	Resolution
**Digital Earth Tester**	Ground resistance	0–20 Ω, 0–200 Ω, 0–2000 Ω	±2% rdg ± 3 dgt (20 Ω & 200 Ω ranges); ± 2% rdg ± 4 dgt (2000 Ω range)	0.01 Ω
**Digital Earth Tester**	Earth Voltage	0–200 V AC (50/60 Hz)	±1% rdg ± 4 dgt	0.1 V

Another method for measuring soil resistivity is the Wenner four-needle method, developed by Dr. Frank Wenner of the United States Bureau of Standards in 1915. Among the various four-needle techniques, Blattner (1982) considered the Wenner configuration the easiest to implement, while other approaches, such as the driven rod test, are more costly and labor-intensive [33]. The method involves inserting four equally spaced electrodes in a straight line, typically one meter apart [34]. A key advantage of this technique is its ability to minimize the influence of surface layers, providing a more representative resistivity measurement of the underlying soil. Researchers have applied the Wenner method to evaluate mixtures of natural reinforcement materials across different soil types. Their findings demonstrated that soil resistivity varies with climatic conditions and the addition of reinforcement materials, resulting in differing resistivity values depending on soil characteristics [35]. Consequently, the four-wire Wenner method is considered a reliable approach for monitoring grounding resistance in homogeneous soils.

Another measurement technique is the clamp-on method, which measures grounding resistance without the need to disconnect or remove the existing grounding system. This method is particularly efficient for systems with pre-installed electrodes. The clamp-on meter operates by inducing a voltage at a specific frequency, typically 1 kHz or 3.4 kHz, and measuring the resulting response. While the method is simple, convenient, and quick to deploy, its accuracy is limited, and it is generally suitable only for loop measurements.

### Grounding electrode installation

Traditional grounding systems are generally designed to achieve resistance values below a specified threshold or according to a defined conductor embedment density. Modern standards, however, focus on the system’s ability to accommodate variations in resistance both within and outside the connected electrical equipment. Over time, various grounding configurations have been developed to further reduce resistance, approaching values near 0 Ω. In low-resistivity soils, grounding is commonly achieved by inserting a copper (Cu) or copper-coated steel rod with a length *L* and radius r into the ground. The resulting grounding resistance, denoted as *R*_*1*_, can be expressed by Equation (1), where *ρ* represents the soil resistivity. These equations are used to calculate the overall resistance in the device.


$$\begin{array}{c} R_{1}=\frac{\rho }{\text{2}\pi L}\left\{\text{ln}\left(\frac{\text{2}L}{r}\left[\text{1}+\sqrt{\text{1}+{\left(\frac{r}{\text{2}L}\right)}^{\text{2}}}\right]\right)\right\}+\frac{r}{\text{2}L}-\sqrt{\text{1}+{\left(\frac{r}{\text{2}L}\right)}^{\text{2}}} \end{array}$$ (1)

$$\begin{array}{cc} R_{2}= & \frac{\rho_{c}}{\text{2}\pi L}\left\{\text{ln}\left(\frac{\text{2}L}{r}\left[\text{1}+\sqrt{\text{1}+{\left(\frac{r}{\text{2}L}\right)}^{\text{2}}}\right]\right)\right\}+\frac{r}{\text{2}L}-\sqrt{\text{1}+{\left(\frac{r}{\text{2}L}\right)}^{\text{2}}}+\frac{\rho -\rho_{c}}{\text{2}\pi L}\left\{\text{ln}\left(\frac{\text{2}L}{d}\left[\text{1}+\;\sqrt{\text{1}+{\left(\frac{d}{\text{2}L}\right)}^{\text{2}}}\right]\right)\right\}+\frac{d}{\text{2}L}-\sqrt{\text{1}}\\ & +{\left(\frac{d}{\text{2}L}\right)}^{\text{2}} \end{array}$$ (2)

$$\begin{array}{cc} R_{3}= & \frac{\rho \cdot \rho_{c}}{\text{2}\pi L\left[H\rho +(L-H)\rho_{c}\right]}\left\{\text{ln}\left(\frac{\text{2}L}{r}\right)\left\{\text{1}+\sqrt{\text{1}+{\left(\frac{r}{\text{2}L}\right)}^{\text{2}}}\right\}\right\}+\frac{r}{\text{2}L}-\sqrt{\text{1}+{\left(\frac{r}{\text{2}L}\right)}^{\text{2}}}\\ & +\frac{H\rho \left(\rho -\rho_{c}\right)}{\text{2}\pi L\left[H\rho +(L-H)\rho_{c}\right]}\times \left\{\text{ln}\left(\frac{\text{2}L}{D}\right)\left\{\text{1}+\sqrt{\text{1}+{\left(\frac{D}{\text{2}L}\right)}^{\text{2}}}\right\}\right\}+\frac{D}{\text{2}L}-\sqrt{\text{1}+{\left(\frac{D}{\text{2}L}\right)}^{\text{2}}} \end{array}$$ (3)

In Equations (1)–(3), *R₁, R₂, and R₃* represent the grounding resistance in (Ω). These are different grounding additive designs with a conventional rod, the rod surrounded by a cylindrical low-resistivity material, and the rod installed within a combined cylindrical-horizontal low-resistivity configuration, respectively. The parameters *ρ* and ρc denote the resistivities of the native soil and grounding enhancement material (proposed additive material) in (Ω·m), respectively. *L* is the length of the grounding rod in (m), *r* is the rod radius in (m), D is the diameter of the low-resistivity material region in (m), *H* is the thickness of the horizontal enhancement layer in (m), and p represents the installation depth parameter in (m). The logarithmic terms remain dimensionless, and the resulting expressions yield grounding resistance in ohms (Ω) [37].

**Fig 2:**
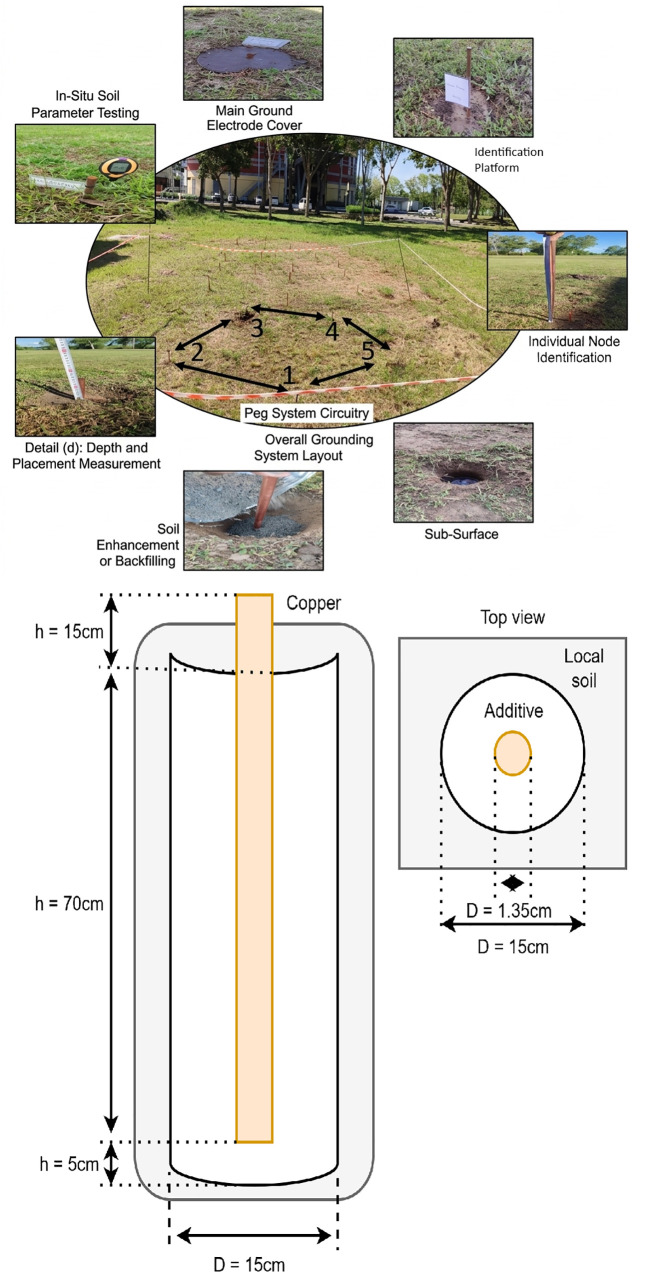
Installation on site, Site location at Miri, Sarawak, approximately 4°19′N latitude and 111°54′E longitude and System insulation dimensions.

To reduce high grounding resistance, low-resistivity materials (LRMs) are often used as backfill [36]. As illustrated in Fig 2, the ground rod is installed in a borehole of appropriate diameter at a depth of 15 cm, after which the LRM is filled into the borehole. The LRM forms a surrounding layer of specific thickness and resistivity, which is itself encased by native soil of uniform high resistivity [37]. The resulting grounding resistance, denoted as *R*_*2*_, can be expressed by Equation (2), where *ρ*_*c*_ represents the resistivity of the second additive material. The installation follows the Code of Practice for Protective Earthing of Electrical Installations (UK), with modifications applied to the rod length as required.

**Fig 3:**
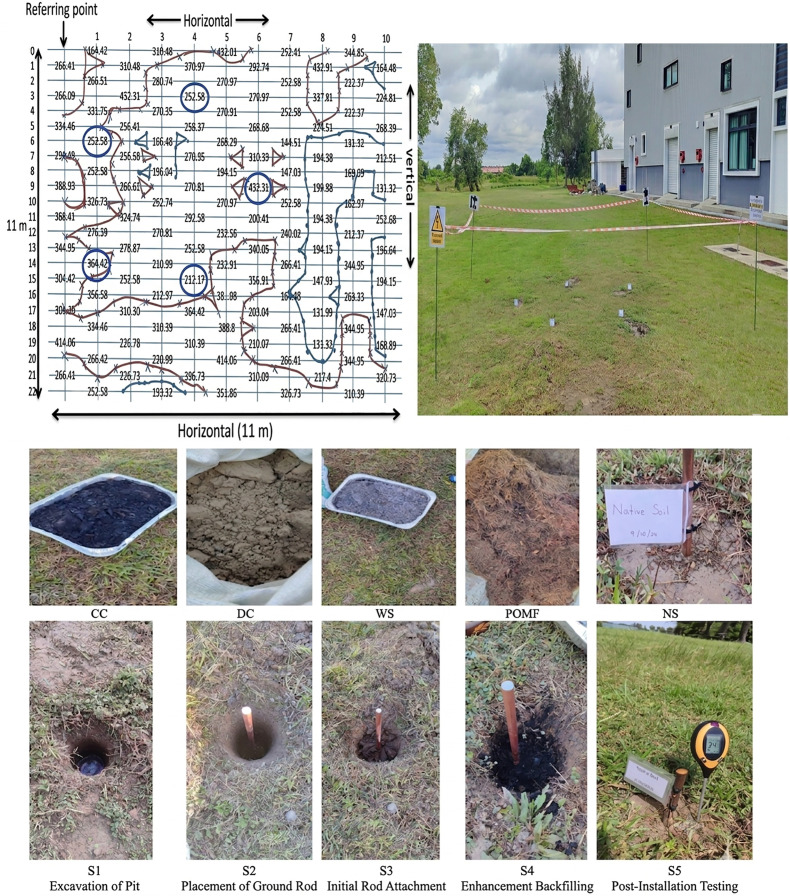
The on-field setup, system pit holes distributions, and procedures for additive filler labeled S1-S5.

As illustrated in Fig 3, steps (S1-S5) inset, this configuration involves digging a hole of appropriate size and placing a vertical ground rod at the center of a horizontal LRM layer with specified diameter and thickness until the end of system installation. This arrangement can significantly reduce grounding resistance [37], with the corresponding resistance, R_*3*_, expressed by Equation (3), where H represents the length of the section composed of the second material, *ρ*_*c*_. This method has been widely applied in experimental studies. For example, one study investigated a concrete grounding system enhanced with bentonite and coconut fiber [38,39]. The experimental setup involved a concrete block measuring 25 × 25 × 30 cm³, installed at a depth of 50 cm, with a copper-plated electrode of 50 cm length and 16 mm diameter. Four concrete mixtures containing different additives were prepared around the ground rod to improve conductivity. The results demonstrated that this approach effectively reduced grounding resistance. The spacing between individual systems was maintained at approximately 1.5 m to prevent underground interactions and ensure isolation between installations. The grounding system pit hole distribution is shown in Fig 3. The point in Fig 3a shows different apparent resistivity *(ρ)* values. Thus, contour lines are constructed based on 200 Ω-m and 300 Ω-m values. Besides that, the location of pit holes will affect the performance of the equipment used during the electrode resistivity measurement. The 200 Ω·m and 300 Ω·m contour levels were selected to describe the dominant low and high-resistivity zones in the site, and the five grounding systems were distributed accordingly to avoid electromagnetic coupling. A center-to-center spacing of approximately 1.5 m was maintained between adjacent systems to reduce overlap of the backfill influence zones and minimize possible mutual interaction during resistance measurements. Although a quantitative electromagnetic coupling analysis was not carried out, the selected spacing was intended to limit interference among the test installations.

The cylindrical pit hole setup is as follows:

For cylinder, *V*_*h*_


$$\begin{array}{c} V_{h}=\pi r^{\text{2}}h \end{array}$$ (4)

*r* = Radius of cylinder

*h* = Height of cylinder


$$\begin{array}{c} V_{h}=\pi (\text{0}.\text{0}\text{7}\text{5}{)}^{\text{2}}(\text{0}.\text{7}\text{0})=\;\text{0}.\text{01236 }{\text{m}}^{\text{3}} \end{array}$$

For Hemi-sphere, V_s_


$$\begin{array}{c} V_{s}=\frac{\text{2}}{\text{3}}\pi r^{\text{3}}(\text{4}.\text{3}) \end{array}$$ (5)

*r* = Radius of hemi-sphere


$$\begin{array}{c} V_{s}=\frac{\text{2}}{\text{3}}\pi {\left(\text{0}.\text{0}\text{7}\text{5}\right)}^{\text{3}}=\;\text{0}.\text{884 x }{\text{1}\text{0}}^{-\text{3}}\;{\text{m}}^{\text{3}} \end{array}$$

Ground Electrode

Pit Hole Total Volume (PHTV) = Volume of cylinder + Volume of hemi-sphere

PHTV = 0.01236 m^3^+ 0.884 x 10^-3^ m^3^ = 0.013244 m^3^

The volume of the ground electrode pit holes is 0.013244 m^3^.

## New biodegradable soil-additive materials

**Fig 4:**
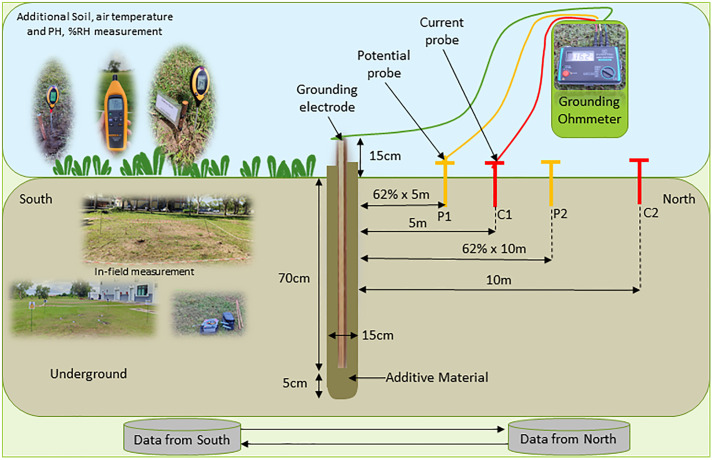
Site measurement setup and data collection using the FOP method.

In the field systems distribution, each point shows a different apparent resistivity, ρ values. Thus, contour lines are constructed based on measured resistivity of the ground and located on 200 Ω-m and 300 Ω-m values as shown in Fig 4a. Otherwise, the location of the pit holes will affect the performance of the equipment used during the electrode resistivity measurement.

Additives such as wood ash (WS), coconut shell charcoal (CC), palm oil mesocarp fiber (POMF), and palm oil filter decanter cake (DC) were incorporated into four grounding systems around the electrodes. For comparison, a reference system was installed in native soil without any reinforcement materials, as shown in Fig 3 (inset NS). This control setup has been widely employed in previous studies, including those reported in [10,13,14,17,21,22,26,32,37]. In the second configuration, CC was used as the backfill material, as shown in Fig 3 (CC inset), offering an alternative method for grounding enhancement [46]. Coconut shells, a hardwood residue, contain lignin, cellulose, and hemicellulose. When subjected to heating, these organic components are converted into carbon-rich products, such as charcoal, which have proven effective for improving grounding system performance [48].

As shown in Fig 3 (POMF inset), the third and fourth test pits were filled with DC in Fig 3 (DC inset), respectively. Both materials are by-products of palm oil production. POMF, also referred to as palm press fiber (PPF), is derived from the biomass residue remaining after the palm fruit is pressed for oil extraction, while palm oil decanter cake is a solid waste generated during the three-phase separation process of palm oil. This paper evaluates these materials not only for their technical performance but also for their economic feasibility and environmental impact, building on earlier studies reported in [26,27]. In this context, the interaction between palm oil waste and peat soil, combined with the presence of a copper electrode, induces biochemical and electrochemical reactions. These collective processes enhance the soil’s electrical conductivity. The chemical reaction for this process can be represented as follows:

Palm oil waste + Soil + Copper.

(*C*_*x*_*H*_*y*_*CO*_*z*_)_Additive material_ + O_2_ + H_2_O → CO_2_ + organic acids (e.g., CH_3_COOH) + H^+^

KCl, NaCl, CaCO_3_ → K^+^ + Na^+^ + Ca^2+^ + Cl- + CO_2_ + H_2_O

Cu → Cu_2_+ + 2e^-^

Cu_2_+ + H_2_S → Cu^2+^ + 2e^+^

This chemical process increases the ionic concentration in the additive surrounding the grounding electrode, thereby enhancing soil conductivity. The final test pit was filled with a mixture of wood ash and sand to achieve the required volume, as wood ash was a limited resource. The sand was sourced from a beach in Miri, while the wood originated from dry pine trees collected locally. When burned, pine wood produces ash primarily composed of oxygen, silicon, calcium, and potassium ions, making it a promising natural material for grounding system reinforcement. Similar approaches have been reported in [10] and [25]. In this study, the backfill mixture consisted of equal parts wood ash and sand (50:50), as shown in Fig 3. The chemical reaction for this process can be represented as follows:

Wood Ash + Soil + Copper

K_2_O, CaO, MgO + H_2_O → 2K^+^+Ca^2+^+ Mg^2+^+2O*H*^–^

Ca(OH)_2_ + CO_2_ → CaCO_3_ + H_2_O

Cu + 2O*H*^–^ → Cu(OH)_2_ → CuO + H_2_O

Both palm oil waste and wood ash positively enhance soil conductivity. Palm oil waste provides an immediate improvement, acting as a biological conductor by releasing ions and moisture; however, its effect diminishes over time as these components are depleted. In contrast, wood ash functions as a mineral conductor, supplying alkaline ions that maintain enhanced soil conductivity over the long term. In this study, the short-term and long-term efficiency of each additive material is systematically investigated.

The selected materials, were chosen because they are locally available waste products generated in significant quantities in Sarawak, Malaysia. These materials are inexpensive, environmentally friendly, and readily accessible. Furthermore, previous studies reported in this paper have shown that has carbon-rich materials, biomass residues, and mineral-rich ashes can improve soil conductivity through moisture retention and ionic transport mechanisms.

## Performance evaluation and enhancement

This part presents the experimental measurement, evaluation, and performance improvement of soil-additive grounding systems under real environmental conditions. The experimental site is located in Sarawak, Malaysia, which experiences a tropical rainforest climate characterized by relatively stable temperatures, high humidity, and substantial annual rainfall throughout the year. As a result, the environmental conditions encountered during the seven-month monitoring period are considered representative of typical local operating conditions. It also covers a post-installation boosting process designed to restore and enhance soil conductivity after performance degradation over time. Collectively, these investigations provide valuable insights into the durability, stability, and reconditioning potential of eco-friendly soil additives for sustainable grounding applications.

### Earth measurement

In this study, the FOP method was employed to comprehensively measure and evaluate grounding performance. This technique is widely recognized for its reliability and applicability in grounding monitoring. The placement of the potential and current probes for resistance measurement followed the 62% rule (0.62 × D, where D is the distance between the ground electrode and the current probe) [14]. This placement minimizes the influence of the electrodes and provides a simple, representative measurement of soil resistivity, even if the soil is not perfectly uniform. The FOP method has been widely adopted in previous studies [10–13,27–32,49], and [50]. Data acquisition was performed using a digital ground resistance tester, which is commonly employed in grounding studies [19,20,38,41], as illustrated in Fig 4.

Several key criteria were followed when conducting the grounding resistance measurements. Two auxiliary electrodes, the current electrode (C) and the potential electrode (P), were positioned in a straight line with the test electrode (grounding electrode). The current electrode was placed 10 meters from the test electrode, while the potential electrode was positioned 6.2 meters away, corresponding to 62% of the total spacing, in accordance with the 62% rule. The test electrode was connected to the green wire, the potential electrode to the yellow wire, and the current electrode to the red wire. To verify measurement consistency, the process was repeated with the auxiliary electrodes repositioned at 5 meters and 3.1 meters, respectively. Measurements were conducted at various distances and orientations to assess soil homogeneity and minimize mutual coupling effects. The ground resistance of each system was measured in the north–south direction, with concurrent recording of soil temperature and pH conditions. The effectiveness of each grounding reinforcement material was then evaluated, considering the measured soil temperature, air humidity (%RH), and ambient temperature.

**Fig 5:**
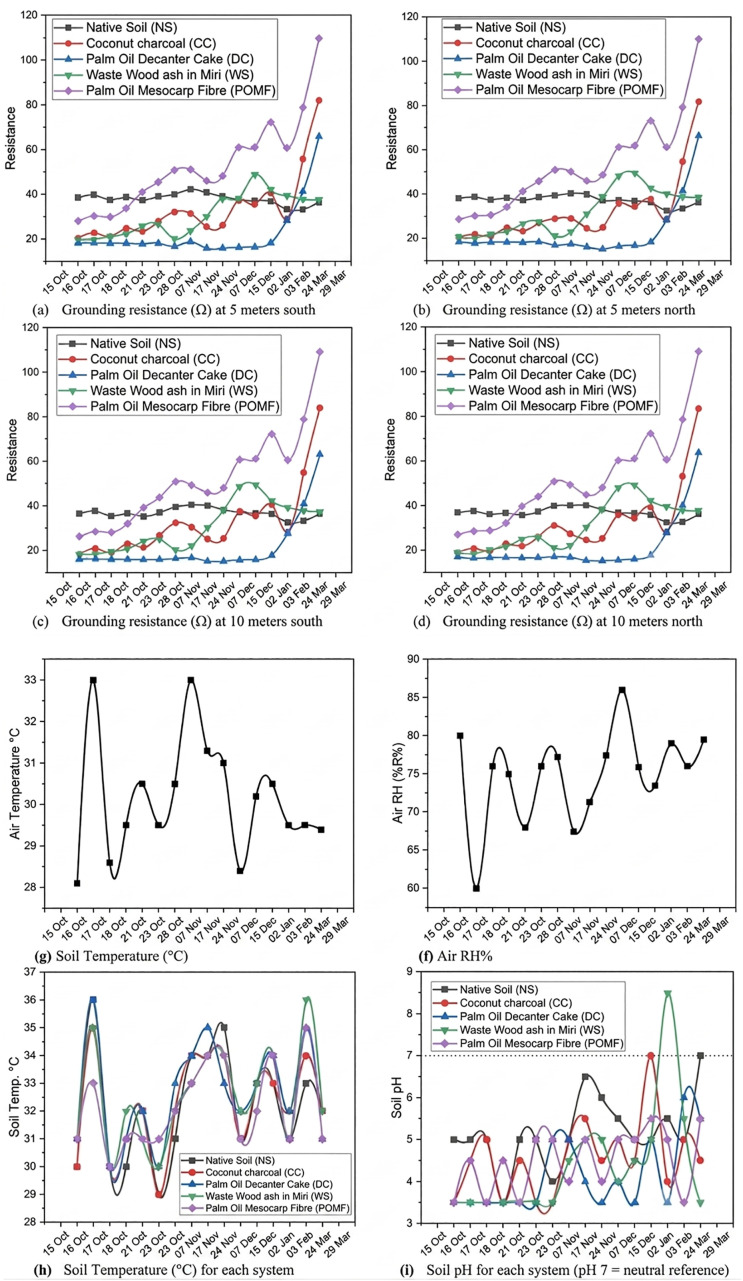
Overall grounding measurements: grounding resistance, ambient temperature, air humidity (%RH), soil temperature, and soil pH level.

The first stage of ground resistance measurements was conducted over 6 months period, from October 15, 2024, to March 29, 2025, using the FOP technique. Alongside resistance measurements, soil and air conditions were continuously monitored. Observations indicated that soil conditions varied between dry on sunny days and moist following rainfall. This variation is critical, as the moisture-retention capacity of a grounding backfill material directly influences its effectiveness; higher moisture retention generally leads to lower ground resistance. The study evaluated and compared the performance of four different soil additive materials incorporated into the proposed grounding system design. The experimental results are presented in Fig 5 illustrate the measured grounding resistances at 5 m and 10 m distances in both the south and north directions, while Fig 5 shows the influence of air temperature and relative humidity (%RH). The effects of soil temperature and soil pH are presented in Fig 5, respectively. Table 3 presents the results, analysis, and discussion of the grounding system measurement period, environmental conditions, resistance behavior, and physical interpretation for each monitoring stage.

**Table 3:** Analysis and discussion of grounding system performance stages.

Months	Measurement details	Grounding System Performance
**1**	Oct 15–29 5:00 PM 7-Tests Sunny days Early performance	Seven resistance measurements were conducted during the monitoring period. As the additive materials stabilized, a gradual decrease in ground resistance was observed, indicating improved homogeneity and ionic conductivity in the surrounding soil medium. All systems incorporating additive materials exhibited lower resistance values compared to the native soil (NS) grounding system during the 7 tests, as shown in Figs 5. However, the POMF-based system demonstrated a more rapid degradation in performance over time, which is attributed to its limited moisture retention capability and faster drying rate.
**2**	Nov 7–24 2 PM – 5 PM 3- Tests Sunny and cloudy Early ageing	The POMF continued to perform poorly, demonstrating its limited suitability as a ground reinforcement material. In contrast, coconut shell charcoal (CC), palm oil decanted cake (DC), and waste wood-soil mixture (WS) exhibited stable behavior and effective resistance reduction during the first two months. Specifically, within this period, CC and DC achieved average resistance values 12.27% and 13.5% lower than the reference system (32.8 Ω), respectively. In comparison, WS and POMF recorded resistance values 21.78% and 86.81% higher than the reference, respectively.
**3**	Dec 7–15 5:30 PM 2- Tests Cloudy Early degradation stage	As shown in Figs 5 and, air temperature gradually decreased while relative humidity increased during the monitoring period. In contrast, both soil temperature and pH exhibited an upward trend. Regarding grounding performance, the DC system maintained stable behavior and continued to demonstrate effective resistance reduction. However, the CC and WS systems showed resistance patterns like the NS system, indicating reduced long-term effectiveness. The POMF system continued to perform poorly, likely due to the decomposition of its organic components, which marked the end of its effective lifespan as a conductive additive.
**4**	Jan 2 – Feb 10 5:30 PM 2- Tests Sunny and cloudy Dry and oxidized stage	DC remained the best-performing additive, attributed to its balanced composition of organic matter and stable ionic content, which supported sustained conductivity over time. WS maintained moderate performance, benefiting from its mineral stability and alkaline nature that helped preserve soil conductivity. In contrast, CC and POMF exhibited rapid performance degradation due to oxidation and moisture loss, leading to a sharp increase in resistivity during the later measurement stages.
**5**	March 7–29 5:30 PM 2- Tests Cloudy and sunny End-of-life observation	At the end-of-life observation stage, the DC and WS materials demonstrated relatively stable performance, maintaining moderate grounding resistance. In contrast, the CC and POMF systems exhibited sharp increases in resistance compared to the NS system. Overall, the results indicate that DC provides both longevity and sustained enhancement of ground conductivity, making it the most effective additive among those tested.
**6**	April 20–30 5:30 PM 5- Tests Cloudy and sunny Boosting soil additive	Based on the above investigations, CC and DC were selected for soil treatment, as both initially exhibited good performance. The results are discussed in the next section.

The reduction in grounding resistance contributed into both electrochemical and physicochemical processes occurring within the soil-additive system. For instance, Coconut shell charcoal (CC) has porous carbonaceous structure which enhance the moisture retention that allows electron transport through interconnected conductive pathways [54]. Also, the palm oil Decanter Cake (DC) contain lignocellulosic materials and residual mineral constituents that improve water-holding capacity. The ESM-EDS for this is presented in [52–53]. This increases the ionic mobility and therefore increases the conductivity. In addition, wood ash contains soluble mineral compounds such as potassium oxide (K₂O), calcium oxide (CaO), magnesium oxide (MgO), and silica (SiO₂). When exposed to soil moisture, these compounds undergo dissolution reactions, releasing ions into the pore water according to reactions such as:

K₂O + H₂O → 2K⁺ + 2OH^–^

CaO + H₂O → Ca²⁺ + 2OH^–^

The released ions increase the electrolyte concentration of the surrounding soil, thereby reducing soil resistivity and facilitating current dissipation from the grounding electrode [25].

Relative to the reference system (34.9 Ω), the resistance of DC increased by 51.86%, CC by 97.13%, WS by 9.06%, and POMF by 170%. This decline was primarily attributed to environmental conditions in March, which reduced soil moisture and affected the effectiveness of the additives. The poor performance of POMF was also linked to its chemical composition: 43% cellulose, 33% hemicellulose, and 22% lignin [51], resulting in limited conductivity and moisture retention. In contrast, the higher carbon content of DC and CC improved conductivity and moisture retention, while wood ash, rich in calcium, potassium, silicon, and oxygen ions [10], initially provided good conductivity but gradually lost effectiveness due to environmental factors and biodegradation [52–54].

Overall, the study evaluated five systems, including four reinforcement configurations (CC, DC, WS, and POMF; WS was prepared in a 50:50 mixture due to limited ash availability) and a reference system. The results indicate that palm oil mesocarp fiber is unsuitable as a ground reinforcement material, whereas CC, DC, and WS show promise. However, further research is required to enhance their long-term durability under varying environmental conditions.

The evolution of grounding resistance throughout the monitoring period can be explained by the combined effects of moisture retention, ionic mobility, and biodegradation of the investigated additives. the conductivity of each additive became increasingly dependent on its ability to preserve moisture and maintain stable conductive pathways. CC and DC provided improved conductivity due to its carbon-rich structure, which enhanced water retention and facilitated charge transport. In contrast, lignocellulosic structure underwent biodegradation in the POMF which reduce its physical integrity and ability to retain moisture. This reduces the continuity of conductive pathways and lowered ionic mobility. The long-term degradation of palm oil mesocarp fibre (POMF) is primarily attributed to microbial decomposition of its lignocellulosic structure, which is rich in cellulose and hemicellulose.

The wood ash and soil mixed WS soluble mineral species such as potassium, calcium, and magnesium ions enhanced conductivity by increasing the ionic concentration of the soil pore water. The overall physical interpretation is shown in Table 4.

**Table 4:** Physical interpretation correlating moisture retention, ionic mobility, biodegradation rate, and conductivity evolution.

Material	Moisture Retention	Ionic Mobility	Biodegradation Rate	Conductivity
**Palm Oil Decanter Cake (DC)**	High	High	Moderate	Most stable, lower resistance
**Coconut Shell Charcoal (CC)**	Initially high	Moderate high	Low	Good, decreases when drying
**Wood Ash and Sand (WS)**	Moderate	High initially due to K ⁺ , Ca² ⁺ ions	Low	Moderate, affected by ion leaching
**Palm Oil Mesocarp Fibre (POMF)**	Low	Low	High	Fast degradation and rapid resistance increase

The statical analysis is performed in this study as shown in Table 5. Statistical analysis confirmed that DC shows the most dependable enhancement in grounding performance. This is by accomplishing an average resistance reduction of 39.94% relative to NS system. The trend slope of 1.39 Ω per measurement interval was the lowest among all additives. The CC and WS shows moderate average reductions of 11.80% and 14.71%, respectively. Although, larger standard deviations indicate 30.95%, and 46.55% greater sensitivity to environmental conditions and ageing. Its trend slope of 1.97 Ω, 2.40 Ω per measurement interval which indicated moderate degradation. In contrast, POMF showed a negative mean reduction (−43.37%), indicating higher resistance than the reference system during much of the monitoring period. POMF exhibited the highest standard deviation (62.68%) and the largest trend slope (3.90 Ω per measurement interval), confirming rapid deterioration throughout the monitoring period. The overall performance ranking can be shown as DC > WS > CC > POMF.

**Table 5:** Statistical summary of grounding resistance performance relative to the Native Soil (NS) system.

Material	Mean Resistance Reduction (%)	Standard Deviation (%)	Trend Slope (Ω per measurement interval)	Reduction Relative to NS Day-0 (%)	Interpretation
**Coconut Charcoal (CC)**	11.80	46.55	2.40	2.44	Gradual degradation
**Palm Oil Decanter Cake (DC)**	39.94	37.28	1.39	57.45	Slower degradation among the additives
**Waste Wood Ash (WS)**	14.71	30.95	1.97	−30.35	Moderate degradation
**Palm Oil Mesocarp Fibre (POMF)**	−43.37	62.68	3.90	−63.41	Fastest degradation

At day 0, the calculation was based on percentage difference. The resistance of the DC system is 57.45% lower than the Native Soil (NS) reference. The CC maintained a resistance level comparable at 2.44% to the Native Soil system, although its resistance increased substantially. The WS and POMF has considerable degradation over time, resulting in final resistance values exceeding those of the reference system. The values WS and POMF resistance increases of 30.35% and 63.41%, respectively, indicating degradation and reduced grounding effectiveness.

### Boosting soil additive measurement

**Fig 6:**
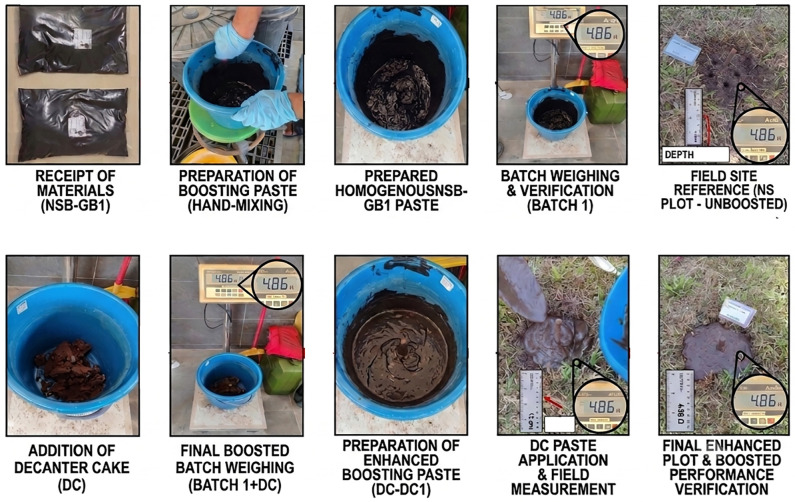
Soil additive boosting process (second stage) for grounding enhancement.

The effectiveness of enhanced grounding systems begins to decline after approximately six months, indicating the need for soil treatment to reduce resistivity and evaluate the duration of restored performance. Common soil treatment methods include the application of salts, water, and carbon-based materials, with salt solutions generally proving more effective at lowering soil resistivity than water or carbon treatment alone [55]. Based on the above investigations, CC and DC were selected for soil treatment, as both initially exhibited good performance but began to decline after six months. For the CC system, activated carbon powder derived from coconut shell charcoal was used. As shown in Fig 6, 1 kg of activated carbon powder was placed in a container, mixed with water, and stirred into a uniform slurry. The powder initially clumped upon contact with water and required thorough mixing to achieve a consistent texture. The resulting slurry was then applied around the grounding system, allowing it to penetrate the soil. A similar approach has been reported in previous studies [28,39], where enhancement solutions were poured around the electrodes.

For the DC system, additional material was applied as a top layer around the electrodes. In this process, 1 kg of DC was mixed with water in a container to form a homogeneous slurry, as shown in Fig 6, and then spread around the grounding system to facilitate absorption by the surrounding soil.

**Fig 7:**
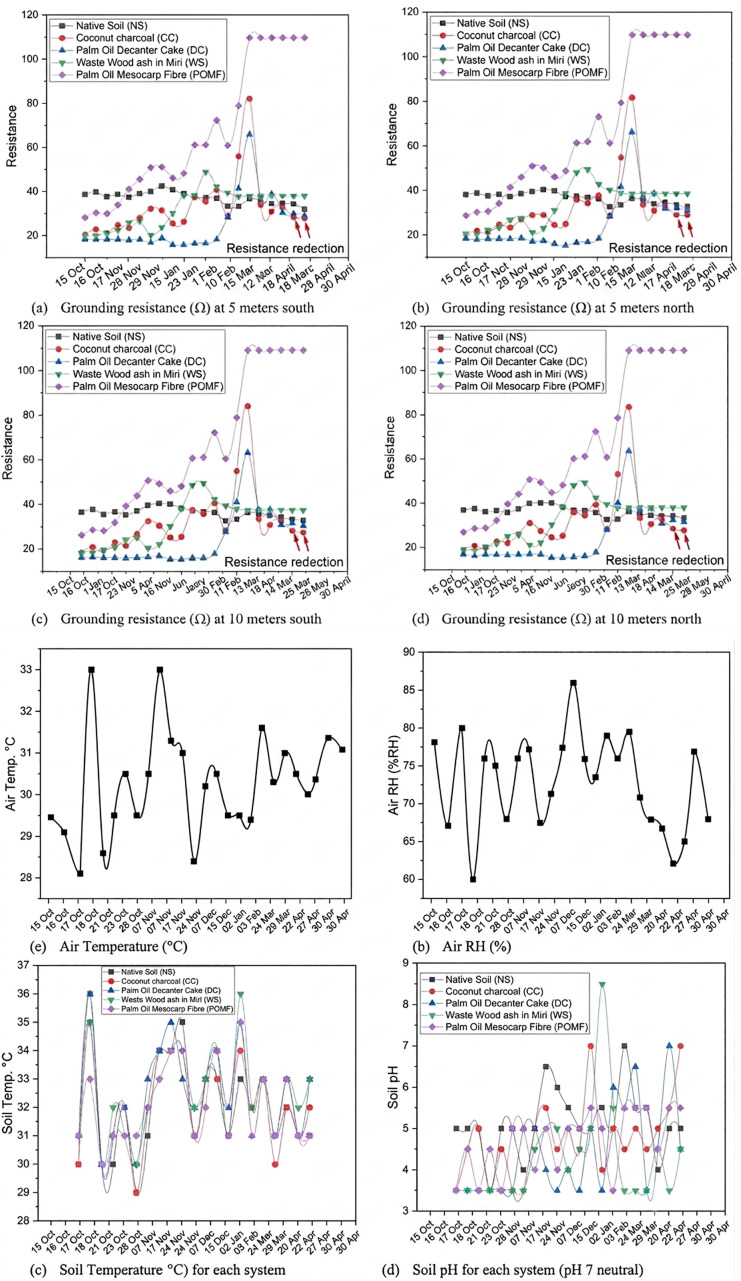
Overall post-grounding measurements: grounding resistance, ambient temperature, air humidity (%RH), soil temperature, and soil pH level.

Before measuring grounding resistance after boosting the additive materials, the treated areas were left to stand for 24 hours to allow the mixtures in both systems to fully absorb into the soil [39]. Resistance measurements were then conducted using the FOP method, while environmental parameters, including air humidity, air temperature, soil temperature, and soil pH, were also recorded. Following established practices, this study monitored the daily resistance values of the grounding systems reinforced with DC and CC. The first reading was taken 24 hours after treatment, and subsequent measurements were recorded over a period from April 2 to April 30, 2025, which indicated between 15 and 25 on the measurement index axis. The effects of the soil enhancement were analyzed and compared, with the results presented in Fig 7.

In the initial phase of post-additive material testing, the resistance of the CC system was lower than that of the reference NS grounding system, while the resistance of the DC system was nearly equivalent to that of the NS system. Over the subsequent days, as the effects of the soil treatment stabilized, the resistance of both systems decreased. After 10 days, the resistance of the CC and DC systems was 15.04% and 5.01% lower than the reference system (33.9 Ω), respectively. Compared to the pre-treatment measurements in Section 4, where CC and DC exhibited resistances 97.13% and 51.86% higher than the reference system, the post-treatment reductions were significant, amounting to 112.17% and 56.87%, respectively. The notable improvement in the CC system can be attributed to the use of activated carbon powder, which enhances electrical conductivity due to its inherently high conductivity (148 *μS*) [56]. The results confirmed that both systems benefited from the soil treatment, with the CC system showing the most significant improvement. Overall, coconut shell charcoal and palm oil decanted cake proved to be effective reinforcement materials for the grounding system. Based on these findings, it is recommended that soil boosting treatments be performed every six months to maintain optimal grounding performance.

The post-installation changes may affect the long-term corrosion behavior of the electrode and the stability of the soil-additive system. While no adverse effects were observed during the present monitoring period, further long-term studies incorporating corrosion measurements, electrochemical analyses, and soil chemistry characterization are recommended to fully assess the sustainability of repeated post-installation treatments.

## Conclusion

This study investigated the effectiveness of waste-derived biodegradable and natural additives for enhancing grounding systems. Five systems monitored for over 6 months using FOP method. The four additive materials listed form higher performance to lower performance are DC, WS, CC, and POMF compared to the reference system (native soil (NS)). The POMF was introduced as a novel material; however, long-term monitoring indicated its inadequacy for grounding enhancement. After approximately five months, the performance of both CC and DC declines after 5 months to 6 months. This recommendation is based on the experimental observations obtained in this study. A post-installation soil treatment was then applied using the same waste-derived materials: activated carbon powder from CC, and DC was mixed with water and applied around the grounding electrodes’ vertical/backfill layer. Both treatments significantly reduced resistance over a two-month measurement monitoring period, confirming the potential of these materials as sustainable, low-cost, and environmentally friendly solutions for grounding enhancement. The study was conducted at a single experimental site under tropical climatic conditions. Before serve as alternative grounding enhancement, full-scale field validation, life-cycle cost assessment, corrosion monitoring, and maintenance optimization to establish their technical and economic feasibility for industrial grounding applications.
